# Acute Calculous Cholecystitis With Sinus Bradycardia: Cope's Sign Encountered

**DOI:** 10.7759/cureus.21187

**Published:** 2022-01-13

**Authors:** Haris Iftikhar, Feroze Salahuddin Khan, Nood Dhafi R Al-Marri, Hany A Zaki, Maarij Masood

**Affiliations:** 1 Emergency Medicine, Hamad Medical Corporation, Doha, QAT; 2 General Practice, Health Education England, Coventry, GBR

**Keywords:** cardio-biliary reflex, bradycardia, gall bladder, biliary colic, cholelithiasis, cholecystitis

## Abstract

Acute cholecystitis and cardiac ischemia can have a similar clinical presentation in some patients. Nonspecific electrocardiographic changes and arrhythmias can occur in acute cholecystitis and gallbladder disease that can confuse the treating physician leading to extensive cardiac workup. Emergency physicians and cardiologists should be aware of these changes so they can expedite the management of acute cholecystitis, which can lead to the resolution of these changes. We report a case of a 53-year-old male who presented with diffuse abdominal pain, nausea, and vomiting. His ECG showed sinus bradycardia. Imaging confirmed the diagnosis of acute calculous cholecystitis. His cardiac workup was unremarkable. His sinus bradycardia was resolved with the management of acute cholecystitis. This case highlights the possibility of a "cardio-biliary reflex" that is initiated by gallbladder pain via autonomic vagal innervation.

## Introduction

Acute cholecystitis and cardiac ischemia can have a similar clinical presentation in some patients. Nonspecific electrocardiographic changes and arrhythmias can occur in acute cholecystitis and gallbladder disease that can further confuse the treating physician, leading to extensive cardiac workup. A vagally mediated cardio-biliary reflex is the proposed cause of some of these changes [1-3]. O'Reilly and Krauthamer were the first to highlight the association between calculous cholecystitis and sinus bradycardia. “Cope's sign” was named after Sir Zachary Cope. He was the first patient and surgeon to report the association of heavy epigastric/central chest pain, attacks of perspiration, and tachycardia with biliary disease [3]. Since the initial publication of two cases, only a few such cases of Cope’s sign have been reported in medical literature, each showcasing some variation of altered heart rhythm in a patient with biliary disease. Due to the paucity of literature on this phenomenon, it is important to report this case where a patient presenting with acute calculous cholecystitis was found to have significant sinus bradycardia at presentation.

## Case presentation

A 53-year-old male presented to emergency medical services at the emergency department (ED), complaining of generalized abdominal pain. The pain was colicky and described as mild but continuous. The onset of pain was one day ago. He had nausea with vomiting three times before presentation. He denied any fever, weight loss, anorexia, or change in bowel or urinary habits. There was no significant past medical or surgical history and family history of cardiac disease. He was not using any medications. He denied smoking and alcohol consumption. He had no known allergies. Physical examination showed a temperature of 37°C, blood pressure of 102/64 mmHg, respiratory rate of 18 breaths/min, pulse rate of 33 beats/min, and saturation of 99% on room air. His respiratory and cardiovascular exam was unremarkable. His abdominal examination showed epigastric tenderness with a negative Murphy’s sign. There was no guarding or rigidity. The rest of his physical exam was also unremarkable.

**Figure 1:**
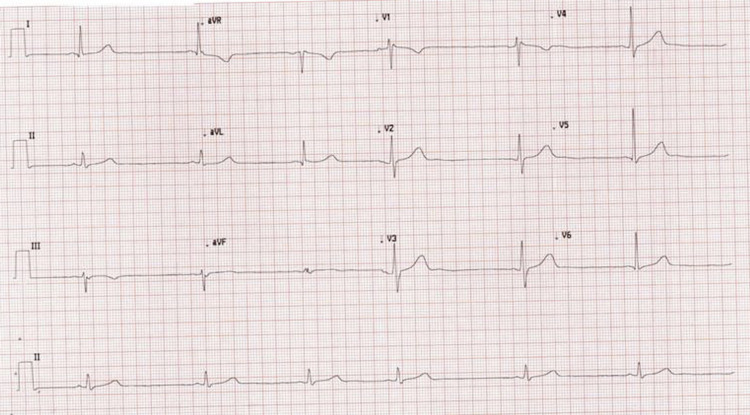
Electrocardiogram (ECG) showing sinus bradycardia and borderline QTc prolongation (QT/QTc: 549/467)

**Figure 2:**
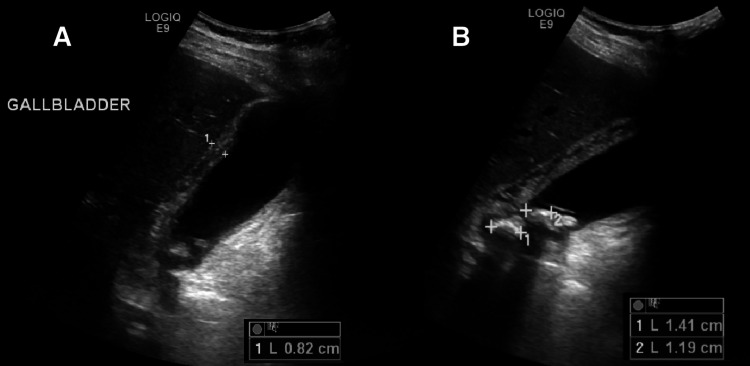
Ultrasound gallbladder showing acute calculous cholecystitis A: The gallbladder (GB) wall is thickened and edematous and measures 8.2 mm. Gallbladder is distended. B: Multiple stones are noted interspersed with sludge. The largest stone measures 14 mm. The rim of pericholecystic fluid is noted around the gallbladder.

An electrocardiogram (ECG) was recorded, which showed sinus bradycardia of 38 beats/min with mild QT interval prolongation (Figure 1). There was no heart block or ST changes. Initial workup was started to evaluate his abdominal pain and to exclude any cardiac pathology. His bedside random blood sugar was 5 mmol/l. Laboratory and radiological investigations (Figure 2) confirmed the diagnosis of acute calculous cholecystitis with high-sensitivity cardiac troponin T assay and thyroid function tests within the normal range. A follow-up ECG again showed sinus bradycardia of 36 beats per minute. Cardiology was consulted to evaluate for his sinus bradycardia and borderline QTc interval prolongation. Cardiology evaluation by Holter monitoring and echocardiography was normal. The patient was admitted under the care of the surgery team and offered emergency laparoscopic cholecystectomy, which he refused. He was managed conservatively and his heart rate was normalized after two days of medical treatment.

## Discussion

Reflex sinus bradycardia due to acute cholecystitis or Cope's sign was first described by O'Reilly and Krauthamer in 1971 [3]. Most cases described in the literature had sinus bradycardia with periods of either complete AV block or sinoatrial exit block as summarized in Table 1. Our case is unique, as this is the third case after the first two cases (Table 1) where there was only sinus bradycardia with acute cholecystitis without any kind of AV or SA exit block. In our literature search, we have identified a total of 10 cases of bradycardia associated with gallbladder disease. Seven were due to acute calculous cholecystitis and one each due to acalculous cholecystitis, biliary colic, and gall bladder torsion. All these cases had resolution of bradycardia with the treatment of gallbladder disease, except one by Scott et al. where ECG findings and resolution were not mentioned [1-11].

**Table 1:** Summary of the findings from the literature review

Year	Authors	ECG Findings	Diagnosis	Resolution of ECG findings
1971	O'Reilly & Krauthamer [3]	Sinus bradycardia (2 patients)	Acute on chronic necrotizing calculous cholecystitis	Yes
1999	Vloka et al. [4]	Severe sinus bradycardia leading to high degree AV block and asystole	Gangrenous acute on chronic calculous cholecystitis	Yes
2009	Franzen et al. [5]	Complete AV block without escape rhythm for 9 seconds	Acute calculous cholecystitis	Yes
2011	Akyel et al. [6]	Idioventricular rhythm with sinus capture beats, sinus bradycardia	Acute calculous cholecystitis	Yes
2015	Lau et al. [7]	Sinus bradycardia with sinus pauses	Acalculous cholecystitis	Yes
2015	Sorić et al. [8]	Complete AV block with a ventricular escape rhythm	Gangrenous acute calculous cholecystitis	Yes
2018	Papakonstantinou et al. [9]	Sinus bradycardia with a brief period of complete AV block	Biliary colic	Yes
2020	Kumar et al. [10]	Sinus bradycardia leading to periods of complete AV block	Acute calculous cholecystitis	Yes
2021	Scott et al. [11]	Bradycardia (ECG not mentioned in the article)	Gall bladder torsion	Not described

It is postulated that a "cardio-biliary reflex" exists that is triggered by gallbladder pain via autonomic vagal innervation. It can occur in cases of gall bladder disease with or without gall stones [7].

## Conclusions

If we consider the patient’s presentation, normal cardiac workup, and resolution of the sinus bradycardia with conservative management of the acute cholecystitis, this patient likely had a case of cardio-biliary reflex. All such cases require special consideration. The altered cardiac rhythm can not only complicate a case of acute cholecystitis but may be part of the initial presentation, thus acting as a confounder to the diagnosis. Further studies may help better delineate this interesting association.
